# Construction and validation of a predictive model for hypocalcemia after parathyroidectomy in patients with secondary hyperparathyroidism

**DOI:** 10.3389/fendo.2022.1040264

**Published:** 2022-11-30

**Authors:** Jingning Cheng, Yong Lv, Ling Zhang, Yafeng Liu

**Affiliations:** ^1^ Department of Otolaryngology-Head and Neck Surgery, China-Japan Friendship Hospital, Beijing, China; ^2^ Department of Nephrology, China-Japan Friendship Hospital, Beijing, China; ^3^ School of Medicine, Anhui University of Science and Technology, Huainan, Anhui, China

**Keywords:** secondary hyperparathyroidism, parathyroidectomy, hypocalcemia, risk factors, nomogram

## Abstract

**Objective:**

We aimed to construct and validate a predictive model for the risk of hypocalcemia following parathyroidectomy (PTX) for the treatment of secondary(renal) hyperparathyroidism (SHPT).

**Methods:**

Information regarding patients with SHPT who underwent PTX between January 2019 and April 2022 was collected retrospectively. Univariate and multivariate logistic regression analyses were used to identify independent risk factors for hypocalcemia following PTX and to construct predictive models. The areas under the receiver operating characteristic curve (AUC), the calibration curve, and the clinical decision curve (decision curve analysis, DCA) were used to assess the discrimination, calibration, and level of clinical benefit obtained using the predictive models.

**Results:**

We studied 238 patients who were randomly allocated in a 7:3 ratio to a training group (n=166) and a test group (n=72). Univariate and multivariate logistic regression analyses were performed, in which three variables (the circulating parathyroid hormone (PTH) and Ca concentrations, and alkaline phosphatase (ALP) activity) were interrogated for possible roles as independent risk factors for hypocalcemia in patients with SHPT who undergo PTX, and used to construct predictive models. The AUCs for the constructed models were high for both the training (0.903) and test (0.948) groups. The calibration curve showed good agreement between the incidence of postoperative hypocalcemia estimated using the predictive model and the actual incidence. The DCA curve indicated that the predictive model performed well.

**Conclusion:**

A predictive model constructed using a combination of preoperative PTH, Ca, and ALP may represent a useful means of identifying patients with SHPT at high risk of developing hypocalcemia following PTX in clinical practice.

## Introduction

Secondary (renal) hyperparathyroidism (SHPT) is a common and serious complication of chronic renal failure that occurs in particular in patients with end-stage renal disease. It results in skeletal lesions and vascular and soft tissue calcification, which seriously affects the quality of life of patients and increases their risks of fracture, cardiovascular events, and death (1). In recent years, with the rapid development of hemodialysis and peritoneal dialysis technologies, the survival time of patients with chronic renal failure has been prolonged and the incidence of SHPT has been increasing (2). The prevalence of SHPT has been estimated to be 20%–80% in patients with chronic kidney disease of various levels of severity (3).

The current treatment of SHPT principally consists of drug therapy and surgery (4). The Global Kidney Disease Guidelines recommend parathyroidectomy for patients with severe hyperparathyroidism who are in chronic kidney disease (CKD) stages 3–5 and in whom drug therapy fails (5). This is an effective method of ablating the lesion and reducing the severity of the symptoms, which include pruritus, bone pain, and bone deformities; and for improving quality of life. However, the surgical removal of the parathyroid glands (PTX) can be associated with complications such as hypoparathyroidism and hypocalcemia. Hypocalcemia is one of the most common complications (6), with a prevalence of up to 20%–85% (7, 8), and can result in convulsions, cardiac arrhythmias, seizures, and even sudden death in patients. The traditional causes of post-PTX hypocalcemia also includes severe vitamin D deficiency (uncorrected hypovitaminosis D) (9), the high frequency of dialysis (7), and the co-presence of malabsorption (10), etc. However, preoperative perioperative preparation may benefit patients more. Therefore, it would be useful to identify preoperative factors that predict the development of hypocalcemia following PTX in patients with SHPT, to guide early clinical intervention and assist in the development of calcium supplementation strategies to prevent the development of severe hypocalcemia.

Previous studies have shown that there are a number of risk factors for hypocalcemia following PTX (8), including age, serum alkaline phosphatase (ALP) activity, number of parathyroidectomy procedures performed, and the circulating parathyroid hormone (PTH) concentration (11, 12). In the present study, we evaluated these clinical factors, plus the preoperative circulating calcium (Ca) and phosphorus (P) concentrations, and the presence or absence of ectopic secretion, for their association with the development of hypocalcemia following PTX in patients with SHPT, to facilitate effective postoperative monitoring and timely and appropriate pharmacological treatment.

## Materials and methods

### Study sample

This is a retrospective study analyzing the clinical data for patients with SHPT admitted to the Department of Otolaryngology-Head and Neck Surgery, China-Japan Friendship Hospital, between January 2019 and April 2022.

The inclusion criteria were: (1) CKD 5-stage patients in regular dialysis, (2) age ≥ 18 years, (3) diagnosis and treatment information were not missing, (4) imaging (ultrasonography, computed tomography, or Technetium-99m methoxy isobutyl isonitrile imaging) results indicative of at least one enlarged parathyroid gland with sharp margins and an intact capsule, and (5) duration of hyperparathyroidism > 6 months. The exclusion criteria were: (1) severe heart, lung, or brain dysfunction prior to surgery, resulting in the inability to tolerate general anesthesia and surgery, (2) impaired cognitive function and communication, (3) diseases that can affect circulating Ca concentration, (4) use of drugs that affect circulating Ca or PTH concentration during the preceding week, such as a calcium-mimicking agent (Cinacalcet), and (5) previous PTX, accompanied by a relapse of SHPT. The study was conducted in accordance with the principles of the Declaration of Helsinki and was approved by the ethics committee of the institution. All the participants provided their written informed consent.

### Study parameters

The clinical characteristics of the participants, including their age and sex, were recorded. Blood samples were collected from all the participants the day before surgery for the quantification of circulating substances, including Ca, P, ALP, and PTH. Total serum Ca concentration was determined using the o-cresolphthalein complex ketone method, serum inorganic P using the phosphomolybdate method, serum ALP activity using an enzymatic method, and intact PTH using a chemiluminescence method. The serum Ca reference range was 1.80–2.75 mmol/L, that for P was 0.97–1.61 mmol/L, that for serum ALP activity was 40–150 U/L, and that for PTH was 12–88 pg/ml. The number of parathyroid glands removed during PTX and whether the tissue was ectopic or not were also recorded. Postoperative blood samples were collected in the morning following surgery, within 48 hours of the procedure. On the basis of the measured postoperative serum Ca concentration, the participants were allocated to two groups: a hypocalcemia group (<1.8 mmol/L) and a non-hypocalcemia group (≥1.8 mmol/L).

### Data analysis

In order to improve and confirm the generalization ability of the prediction model, and avoid the existence of over-fitting or under-fitting. The population was divided into 7: 3 groups with the random seed number of 11. 70% were used as the training group to build the model. 30% as the test group, used for model performance evaluation.

Empowerstats (http://www.empowerstats.com) was used for statistical analysis. Continuous variables are expressed as mean ± standard deviation, and Student’s *t*-test or One-Way ANOVA were used to compare the groups. Categorical variables were compared using the χ^2^ test, and are expressed as frequencies and ratios. Logistic regression analysis was used to identify independent risk factors. The “pROC” package was used to plot receiver operating characteristic (ROC) curves and the “RMDA” package was used to plot DCA clinical benefit curves. The “RMS” package was used to plot calibration curves and nomograms. P < 0.05 was considered to be statistically significant.

## Results

### Characteristics of the participants

A total of 238 patients with SHPT were included and randomly allocated to training and test groups in a ratio of 7:3 (n=166 in the training group: 61 that experienced postoperative hypocalcemia and 105 that did not; and n=72 in the test group: 27 that experienced hypocalcemia and 45 that did not) (**Table 1**).

**Table 1:** Description of study population.

	Train group	Test group	Standardize diff.	P-value
**N**	166	72		
**AGE**	47.139 (11.457)	46.333 (10.143)	0.074 (-0.202, 0.351)	0.607
**PTH**	1672.633 (958.146)	1496.810 (1050.398)	0.175 (-0.102, 0.452)	0.208
**CA (preoperative)**	2.474 (0.286)	2.454 (0.290)	0.068 (-0.208, 0.345)	0.627
**P**	2.003 (0.569)	1.915 (0.700)	0.138 (-0.139, 0.415)	0.308
**ALP**	423.355 (524.603)	339.403 (459.554)	0.170 (-0.107, 0.447)	0.241
**GENDER**			0.149 (-0.128, 0.426)	0.292
female	73 (43.976%)	37 (51.389%)		
male	93 (56.024%)	35 (48.611%)		
**Number of resections**			0.174 (-0.103, 0.451)	0.224
1-3	62 (37.349%)	21 (29.167%)		
≥4	104 (62.651%)	51 (70.833%)		
**Ectopia plant**			0.084 (-0.193, 0.361)	0.554
no	62 (37.349%)	24 (33.333%)		
yes	104 (62.651%)	48 (66.667%)		
**hypocalcemia**			0.016 (-0.261, 0.292)	0.912
no	105 (63.253%)	45 (62.500%)		
yes	61 (36.747%)	27 (37.500%)		

### Results of the univariate and multivariate analyses of hypocalcemia following PTX in patients with SHPT

Univariate and multivariate logistic regression analyses were performed to identify risk factors for postoperative hypocalcemia in 166 patients with SHPT. The parameters for which *P*<0.05 was calculated in univariate logistic regression analysis (age, PTH, Ca, and IP) were included in multivariate logistic regression analysis. The results of this analysis identified PTH, Ca, and ALP (*P*<0.05) as independent risk factors for hypocalcemia following PTX in patients with SHPT (**Table 2**).

**Table 2:** Univariate and multivariate logistic regression analysis.

	Univariate			Multivariate	
	Statistics	OR (95%CI)	P-value	OR (95%CI)	P-value
**GENDER**					
female	73 (43.976%)	ref			
male	93 (56.024%)	1.674 (0.876, 3.200)	0.119		
**AGE**	47.139 ± 11.457	0.955 (0.927, 0.984)	0.002	0.980 (0.943, 1.019)	0.310
**Number of resections**					
1-3	62 (37.349%)	ref			
≥4	104 (62.651%)	1.220 (0.632, 2.356)	0.553		
**Ectopia plant**					
1-3	62 (37.349%)	ref			
≥4	104 (62.651%)	0.874 (0.457, 1.675)	0.686		
**PTH**	1672.633 ± 958.146	1.001 (1.001, 1.002)	<0.001	1.001 (1.000, 1.002)	<0.001
**Ca (preoperative)**	2.474 ± 0.286	0.021 (0.004, 0.096)	<0.001	0.017 (0.002, 0.115)	<0.001
**P**	2.003 ± 0.569	1.130 (0.647, 1.973)	0.669		
**ALP**	423.355 ± 524.603	1.003 (1.002, 1.005)	<0.001	1.001 (1.000, 1.003)	0.049

### Construction and validation of a predictive model

**Figure 1:**
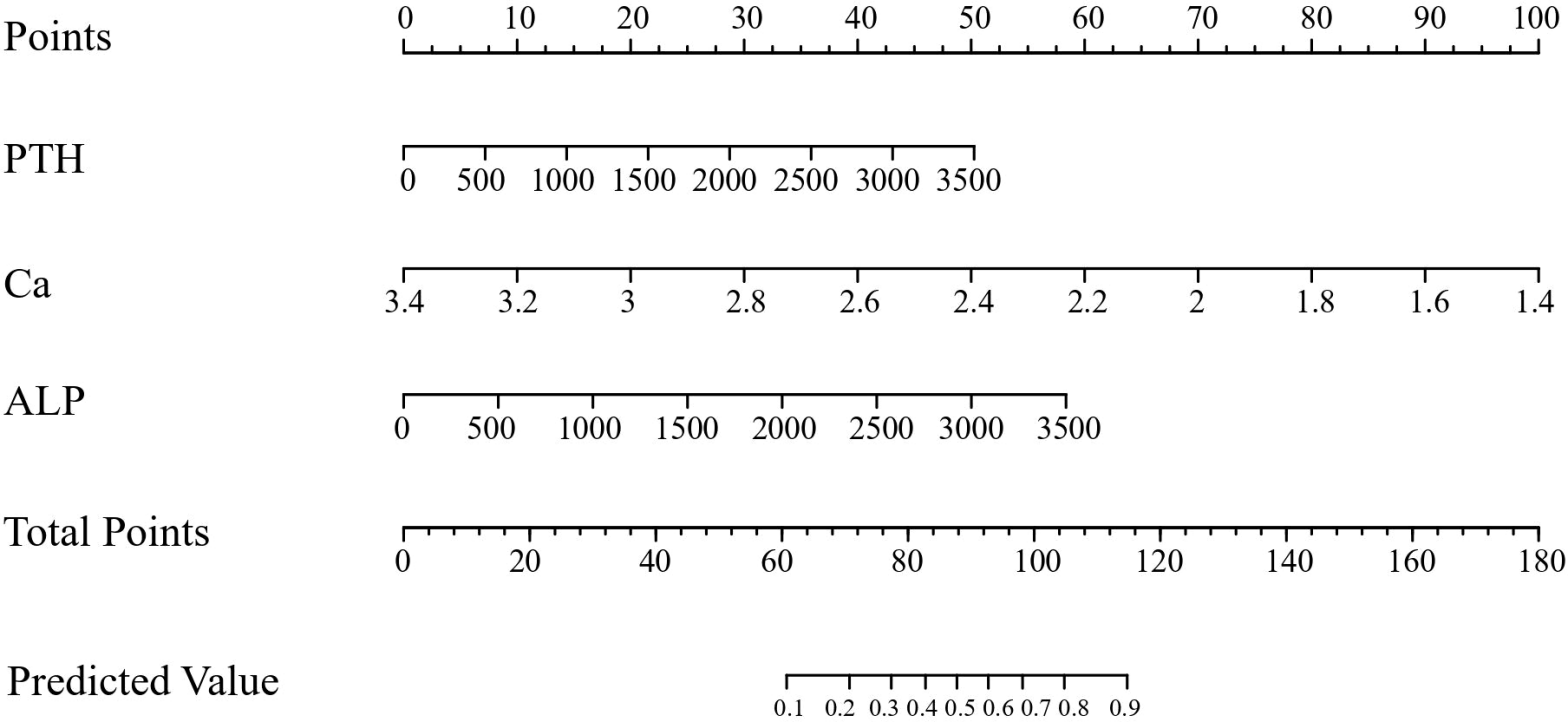
Nomogram for the predictive model.

PTH, Ca, and ALP were used as independent variables and the occurrence of hypocalcemia was used as a dependent variable to construct a mathematical prediction model: Logit (p) = 5.16568 + 0.00134 * PTH-3.44615 * CA+0.00136 * ALP.To increase the usefulness of this model, we generated a nomogram that shows scores corresponding to each risk factor and a total of all the risk factors, corresponding to the risk of postoperative hypocalcemia (**Figure 1**).

The application of nomogram was as follows: According to the figure, the score value corresponding to each prediction index could be found and recorded as the total score. The prediction probability corresponding to the total score in the last row of the figure was the risk of hypocalcemia (range 0–1).

**Figure 2:**
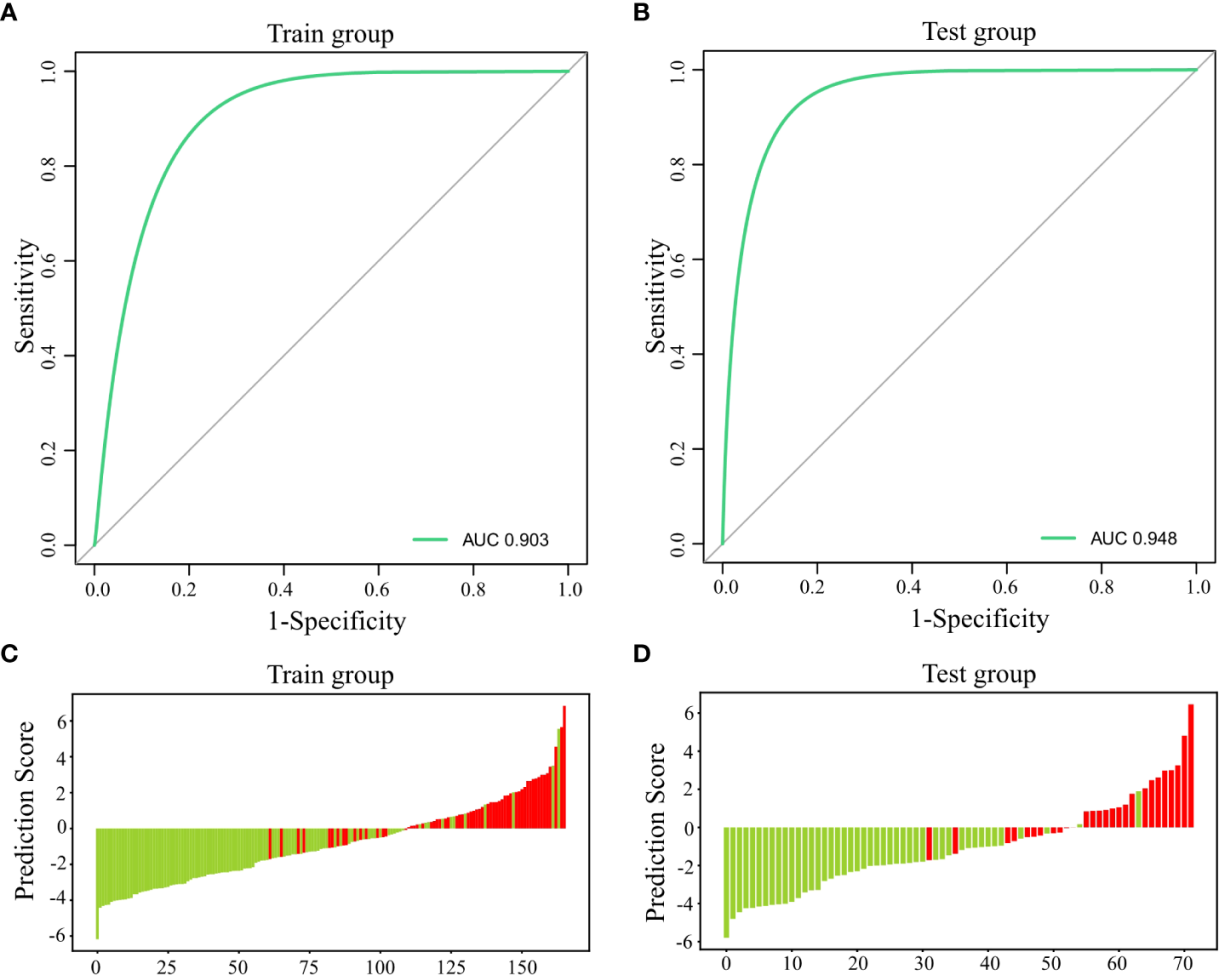
Predictive efficiency and score for the predictive model. **(A)** AUC for the training group; **(B)** AUC for the test group; **(C)** Predictive score for the training group; and **(D)** Predictive score for the test group.

A ROC curve was plotted using PTH, Ca, and ALP for the training group, which yielded an AUC of 0.903 [95%CI: 0.856-0.945, cutoff value: 66.9%] (**Figure 2A**). The same model was used for the ROC analysis of data from the 72 participants in the test group, which yielded an AUC of 0.948 [95%CI: 0.845-0.97] (**Figure 2B**).

The predictions for the participants in the training and test groups are shown using a green line for non-hypocalcemic participants and a red line for hypocalcemic participants (**Figures 2C, D** **)**, which demonstrates a clear separation of the two groups.

### Calibration and clinical benefit of the predictive model

**Figure 3:**
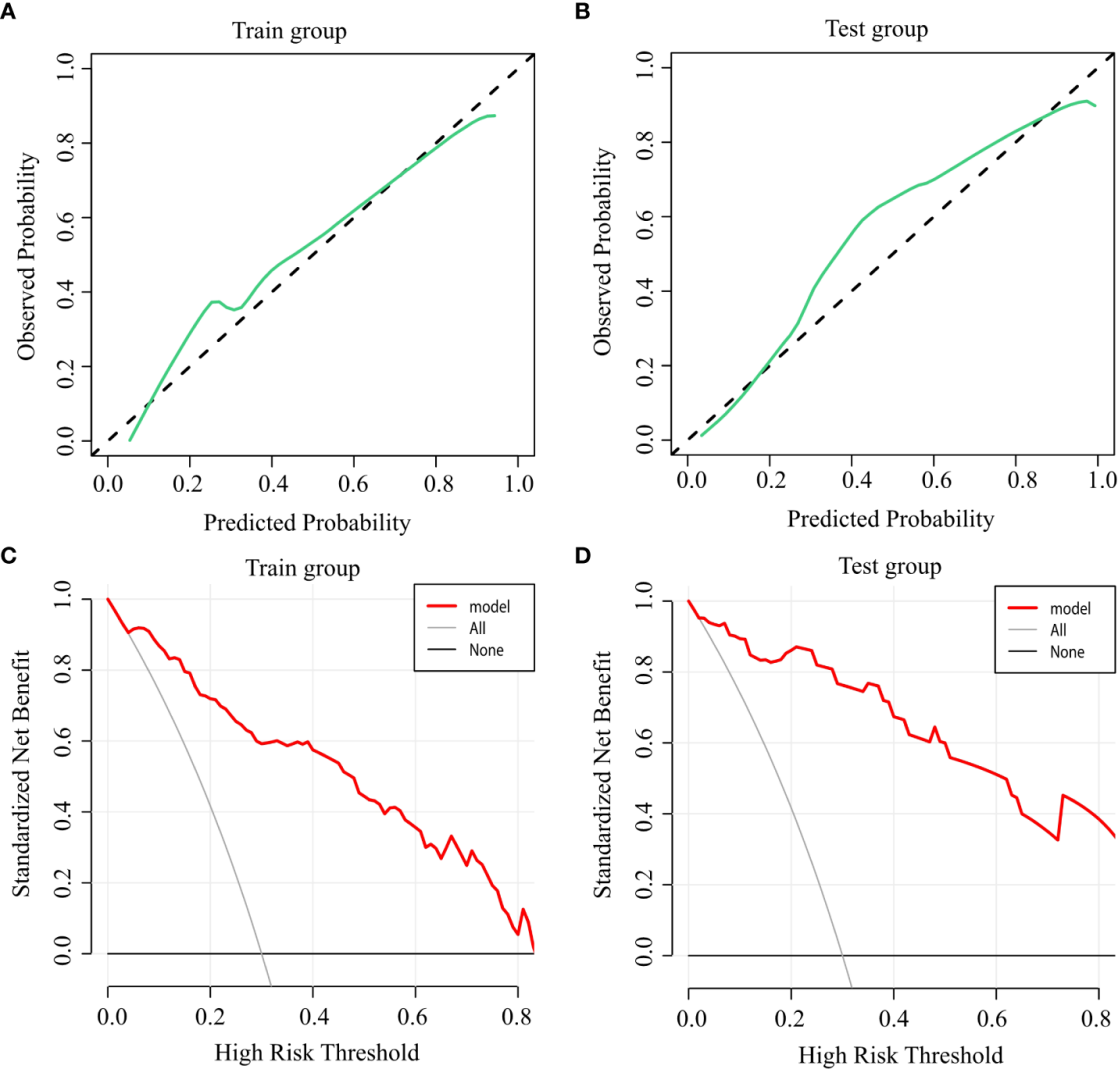
Accuracy and clinical benefit of the predictive model. **(A)** Calibration curve for the training group; **(B)** Calibration curve for the test group; **(C)** Decision curve for the training group; and **(D)** Decision curve for the test group.

Calibration graphs were plotted, with green lines representing the standard curves for the predictive model and black dashed lines representing the calibration curves. Hosmer-Lemeshow goodness-of-fit test was performed on the prediction model: training group: X^2^ = 9.927, P=0.270 (**Figure 3A**); test group: X^2^ = 2.227, P=0.973 (**Figure 3B**). The calibration curve is consistent with the rational curve, and the difference between the predicted value and the observed value has no systematic significance, indicating that the model is a reliable means of predicting the risk of hypocalcemia following PTX in patients with SHPT.

DCA was used to evaluate the clinical applicability of the predictive model, when the threshold probability was within the range of 5%–88%, the net benefits of patients were higher than the two extreme curves in the figure, indicating that the range had clinical effectiveness. The cutoff value of 66.9% obtained by ROC analysis of the above figure was also within the threshold probability of the clinical decision curve of the prediction model, so it could be seen that the prediction model was clinically effective. Further analysis shows that if the risk of hypocalcemia calculated by the prediction model is greater than 66.9%, the corresponding intervention measures will be taken, resulting in a net benefit rate of 35% in the modeling population (**Figure 3C**) and 38% in the verification population (**Figure 3D**).

## Discussion

SHPT is a common complication in patients with end-stage renal disease (13). High serum PTH concentrations cause the mobilization of bone calcium and abnormal deposition of calcium and phosphorus in soft tissues, such as blood vessels, skin, and heart valves, leading to osteoporosis, bone pain, fractures, severe vascular sclerosis, and pruritus, and thus a poor prognosis (3, 14). The early stages of SHPT can be controlled using drugs, including vitamin D receptor activators and cinacalcet (15), but as the symptoms worsen and drug therapy becomes ineffective, PTX is frequently performed (16). Hypocalcemia is a common complication of PTX, manifesting as a sudden reduction in serum PTH concentration following surgery, but a small amount of PTH remains in the circulation. At this stage, a small dose of PTH causes an increase in osteoblast activity, greater bone formation, the redeposition of calcium and phosphorus in the bone, and decreases in the serum calcium and phosphorus concentrations (17, 18).

Hypocalcemia causes numbness of the patient’s hands and feet, and as it worsens, hand and foot convulsions, and tracheal spasm and death by asphyxiation can occur. These problems are associated with significant damage to the patient’s mental, physical, and economic well-being. Previous studies have shown that the incidence of postoperative hypocalcemia in patients with SHPT can be as high as 70%–90% (11, 19), and postoperative hypocalcemia usually occurs on postoperative day 1. Therefore, close collaborative monitoring and postoperative management by nephrologists, otolaryngologists, and thyroid surgeons is required. Specifically, the early screening of patients at high risk for hypocalcemia following PTX should be performed to prevent severe hypocalcemia and provide more timely and effective treatment and postoperative management.

The associations of eight factors (age, sex, PTH, Ca, IP, ALP, the number of parathyroid glands resected, and the presence of ectopic parathyroid tissue) with the development of hypocalcemia following PTX in patients with SHPT were assessed in the present study. We found that PTH, Ca, and ALP were predictors of hypocalcemia following PTX in patients with SHPT. Circulating biochemical substances can be sensitive and/or specific indicators of pathological and physiological changes, and can be used as safe, non-invasive, and reproducible assays for the diagnosis and/or prevention of diseases (20). Calcium ions, which are essential for homeostasis, maintain the biopotential cell membranes, and are important for neuronal signal conduction, muscle contraction, and diastolic function (21). Serum calcium concentration is closely related to the secretory function of the parathyroid glands, and is directly measured to monitor patients for hypocalcemia following PTX (22). In the present study, low preoperative serum Ca concentration was found to be an independent risk factor for postoperative hypocalcemia, and several previous studies have generated similar results (11, 23).

PTH is a basic single-chain polypeptide hormone that is secreted by parathyroid principal cells (24) and increases the activity and number of osteoclasts, thereby inducing an increase in circulating calcium concentration (25), as well as inhibiting phosphorus absorption by the renal tubules. In patients with SHPT, the excessive secretion of PTH leads to bone mineral loss and bone malnutrition. Following PTX, osteoclast activity is reduced and osteoblast activity is not significantly inhibited; therefore, the rate of bone formation exceeds that of resorption, and calcium is transferred from the circulation to bone (26). A high pre-operative PTH concentration results in severe bone malnutrition and substantial calcium deposition in the bone following PTX, causing severe hypocalcemia, which at least in part explains the relationship between post-operative calcium concentration and PTH. The present findings are consistent with those of several previous studies (27, 28), in which a high preoperative PTH concentration was found to be a predictor of postoperative hypocalcemia.

Serum ALP has 4 sources: liver, bone, placenta, and intestine, and ALP is principally released by the intestine after eating a large amount of fat (29). In the present study, liver disease, pregnancy, and the intestine were excluded as sources of ALP, suggesting that high serum ALP concentrations were caused by abnormal bone metabolism, and are principally accounted for by increases in bone-specific ALP (BALP) activity, which reflects bone metabolism more accurately than total ALP. BALP is a glycoprotein synthesized by osteoblasts that hydrolyzes pyrophosphate to produce phosphate during bone mineralization and contributes to hydroxyapatite precipitation. If bone mineralization is disturbed, osteoblasts synthesize more BALP as a negative feedback response (30), which may explain the high preoperative serum ALP activities identified in patients at high risk of hypocalcemia.

Consistent with the present findings, it has previously been shown that patients with high preoperative ALP activities are at higher risk of postoperative hypocalcemia (31, 32). A study by Yang et al. (33) also showed that patients with preoperative serum ALP activities > 277 U/L are susceptible to postoperative hypocalcemia, but the clinical utility of ALP alone for the prediction of hypocalcemia was unsatisfactory in this study, whereas the combined index identified in the present study is a more accurate predictor.

In the present study, we identified preoperative PTH, Ca, and ALP as risk factors for severe hypocalcemia during the early postoperative period. Given the mechanism whereby hypocalcemia develops soon after surgery in patients with SHPT, it is likely that the preoperative PTH concentrations of the hypocalcemic group were higher, which would explain the severe bone starvation state in this group. Indeed, the higher the preoperative PTH concentration of the patient, the more severe is their hyperparathyroidism, and the greater their preoperative bone resorption. However, the lower their PTH concentration is following surgery, the greater is their calcium and phosphorus deposition in bone, and the more likely it is that hypocalcemia will occur.

In this study, when the cutoff value of 66.9% of the ROC curve was used as the threshold probability value of the DCA curve, the clinical net benefit rate of the patient was higher than that of the two extremes of no or all intervention, which also suggested that when the model predicted that the risk of hypocalcemia in patients was higher than 66.9%, immediate intervention would benefit patients clinically. When the cutoff value was lower than 66.9%, the occurrence of hypocalcemia could be dynamically observed.

Nevertheless, the present study had some limitations. The number of potential risk factors in this model is limited. Because it was a retrospective study, there may have been some selection bias, and therefore prospective cohort studies should be performed in the future to test the veracity of the present findings.

In conclusion, the combination of preoperative low Ca, high ALP, and high PTH has a good predictive value for hypocalcemia following PTX in patients with SHPT. The predictive model constructed in the present study should assist clinicians to fully assess the risk factors present in patients prior to surgery, and therefore help them minimize the risk of postoperative complications.

## Data availability statement

The raw data supporting the conclusions of this article will be made available by the authors, without undue reservation.

## Ethics statement

The studies involving human participants were reviewed and approved by China-Japan Friendship Hospital. The patients/participants provided their written informed consent to participate in this study. Written informed consent was obtained from the individual(s) for the publication of any potentially identifiable images or data included in this article.

## Author contributions

JC and YL performed all the surgeries, YL and LZ proposed the topic, JC wrote the paper, YFL conducted the statistical analysis. All authors contributed to the article and approved the submitted version.

## Acknowledgments

We thank Mark Cleasby, PhD from Liwen Bianji (Edanz) (www.liwenbianji.cn) for editing the language of a draft of this manuscript.

## Conflict of interest

The authors declare that the research was conducted in the absence of any commercial or financial relationships that could be construed as a potential conflict of interest.

## Correction note

A correction has been made to this article. Details can be found at: 10.3389/fendo.2025.1777571.

## Publisher’s note

All claims expressed in this article are solely those of the authors and do not necessarily represent those of their affiliated organizations, or those of the publisher, the editors and the reviewers. Any product that may be evaluated in this article, or claim that may be made by its manufacturer, is not guaranteed or endorsed by the publisher.
