# Correction: Construction and validation of a predictive model for hypocalcemia after parathyroidectomy in patients with secondary hyperparathyroidism

**DOI:** 10.3389/fendo.2025.1777571

**Published:** 2026-01-12

**Authors:** Jingning Cheng, Yong Lv, Ling Zhang, Yafeng Liu

**Affiliations:** 1 Department of Otolaryngology-Head and Neck Surgery, China-Japan Friendship Hospital, Beijing, China; 2 Department of Nephrology, China-Japan Friendship Hospital, Beijing, China; 3 School of Medicine, Anhui University of Science and Technology, Huainan, Anhui, China

**Keywords:** secondary hyperparathyroidism, parathyroidectomy, hypocalcemia, risk factors, nomogram

In the published article, there was an error in the author list. Authors “Jingning Cheng” and “Yong Lv” were not identified as co-first authors. A footnote to the author list has been included: “These authors share first authorship”.

